# Training multidisciplinary practitioners to deliver SBI on alcohol and other health behaviours

**DOI:** 10.1186/1940-0640-8-S1-A26

**Published:** 2013-09-04

**Authors:** Niamh M Fitzgerald, Susie Heywood

**Affiliations:** 1 Create Consultancy Ltd., Glasgow, Scotland, UK; 2 Robert Gordon University, Aberdeen, Scotland, UK

## 

We describe our experiences developing a model of delivery and multidisciplinary training course for screening and brief advice on four lifestyle issues: alcohol, smoking, physical activity, and diet. The one-day, 6-hour training course was developed to equip a wide range of health, social care, and community practitioners with an understanding of basic principles of screening and brief advice and key public health messages on the four lifestyle issues. The course was framed around an overall flow-diagram to illustrate a suggested process of brief advice including raising lifestyle issues and choosing (with the patient/client) whether to focus on one or more issues. The training utilised a series of interactive activities focusing on attitudes and skills including individual, paired, triad and group elements. A training for trainers course was delivered over 3 days to practitioners including prison officers, nurses, health trainers, fitness trainers, dieticians and others. In reflecting on the process and feedback from participants we feel:

• It is challenging to train practitioners on SBI generally, and harder to ensure implementation, so it remains to be seen how effective this approach will be.

• Covering four topics together can be seen by funders as a more efficient (or cheaper) way to spend public health budgets.

• This is risky, as it can be difficult for practitioners to absorb information about four topics at once.

• Based on previous experiences in Scotland, we can also speculate that practitioners may not consistently or objectively choose which lifestyle issue to focus in discussion with patients/clients and may avoid topics seen as more complex or sensitive such as alcohol or diet.

